# Exploration of Sp-Sp^2^ Carbon Networks: Advances in Graphyne Research and Its Role in Next-Generation Technologies

**DOI:** 10.3390/ijms26115140

**Published:** 2025-05-27

**Authors:** Muhammad Danish Ali, Anna Starczewska, Tushar Kanti Das, Marcin Jesionek

**Affiliations:** 1 Institute of Physics Centre for Science and Education, Silesian University of Technology, Krasińskiego 8, 40-019 Katowice, Poland or muali@polsl.pl (M.D.A.); tushar.kanti.das@polsl.pl (T.K.D.); 2 PhD School, Silesian University of Technology, 2a Akademicka Str., 44-100 Gliwice, Poland

**Keywords:** graphyne, structure, α-graphyne, β-graphyne, γ-graphyne, applications

## Abstract

Graphyne, a hypothetical carbon allotrope comprising sp and sp^2^ hybridized carbon atoms, has garnered significant attention for its potential applications in next-generation technologies. Unlike graphene, graphyne’s distinctive acetylenic linkages endow it with a tunable electronic structure, directional charge transport, and superior mechanical flexibility. This review delves into the structural variety, theoretical underpinnings, and burgeoning experimental endeavors associated with various graphyne allotropes, including α-, β-, γ-, and 6,6,12-graphyne. It examines synthesis methods, structural and electronic characteristics, and the material’s prospective roles in diverse fields, such as nanoelectronics, transistors, hydrogen storage, and desalination. Additionally, it highlights the use of computational modeling techniques—density functional theory (DFT), GW approximation, and nonequilibrium Green’s function (NEGF)—to anticipate and validate properties without fully scalable experimental data. Despite substantial theoretical progress, the practical implementation of graphyne-based devices faces several challenges. By critically assessing current research and identifying strategic directions, this review underscores graphyne’s potential to revolutionize advanced materials science.

## 1. Introduction

Men have developed various techniques to make life easier and find new ways to produce energy. This era is known as the modern era, and a lot of new technologies have been developed to check the performance of materials. There are a lot of materials that exhibit higher values against various properties, such as thermal [1], electrical [2], dielectric [3], electronic [4], sensing [5], storage [6], antimicrobial [7], etc. Carbon and carbon-based materials are promising materials for the above-mentioned properties. The presence of various states of hybridization (sp, sp^2^, sp^3^) results in the formation of different carbon allotropes with diverse covalent bonds between carbon atoms. Diamond and graphite are the most natural allotropes of carbon, and they have hybridization states of sp^2^ and sp^3^, respectively. Carbon is present in almost every walk of life and provides the basis of life on Earth. Materials scientists are exploring new ways of synthesizing (theoretically and experimentally) carbon allotropes to discover new carbon phases with unique properties and bonding with higher stability [8]. In 1987, Baughman first published a report on accidentally forming a new form of carbon [9]. After the discovery of graphene (G) [10] in 2004, many researchers explored its various properties that are beneficial for the applications of electronics: EMI shielding, supercapacitor, electrical, dielectric, antibacterial, sensing, etc. [11]. After this, in 2010, Li et al. worked on Cu cross-coupling with GO and found GDR with high surface area [12]. New methodologies were searched out for the synthesis of GDR [13]. On ZnO nanorod arrays, various thicknesses of GDR were synthesized [14]. Using the eco-friendly synthesis method, GDR can be synthesized in the form of nanowalls, nanotubes, and nanowires [15,16,17,18]. The GDR shows manifesting results in ion batteries, solar cells, catalysis, and water purification applications [19,20,21,22,23,24,25,26].

With a honeycomb lattice structure [27], G is known as the strongest material among all known elements with a tensile strength greater than 100 GPa, and a tensile modulus of 1 TPa [28]. Due to its higher conductivity and mechanical properties, in comparison with other materials that have 2D structures in hexagonal lattices [29,30,31], G showed some manifesting results of electrical, mechanical, storage, EMI shielding properties, etc. [32,33,34,35,36,37,38,39,40,41,42,43,44,45,46,47,48]. After the discovery of GDR, it caught the attention of scientists because of its thermal, mechanical, structural, and electronic properties [49,50,51]. As novel forms of allotropes of carbon, GO, graphyne (GR), graphdiyne (GDR), and G are considered the best materials for various applications of intriguing electronic and mechanical properties, optical energy storage, and promising nanoelectronics [52,53,54,55,56,57].

Due to this property, carbon can be synthesized in the form of nanorods, G, carbon nanotubes (CNT), nanobuds, fullerenes, nanorings, etc. [8,10,58,59,60,61,62,63,64,65]. Synthesis of fullerene [61], CNT [62], and G are one of these [10]. Baughman et al. [9] proposed a new family member of carbon-based materials, GR, which consists of sp^2^ and sp carbon atoms with one atom thickness, by replacing the aromatic bond with an acetylenic group (-C≡C-) in G to produce GR [55]. After the concept of GR, several researchers tried to synthesize and find the electronic properties of the GR [55,66,67,68,69].

The search for advanced materials with exceptional properties has led to the continuous evolution of carbon-based structures, renowned for their diverse hybridization states (sp, sp^2^, and sp^3^) and broad functional versatility. While natural allotropes like graphite and diamond are well-known, synthetic carbon materials—such as fullerenes, carbon nanotubes, graphene, and more recently, graphyne—have opened new directions in materials science. Among these, graphene has gained immense attention due to its outstanding mechanical strength, high electrical conductivity, and zero-band-gap semimetallic behavior. However, its lack of an intrinsic band gap limits digital electronic applications requiring precise on/off switching.

Graphyne, a theoretically predicted two-dimensional carbon allotrope composed of sp and sp^2^ hybridized carbon atoms, has emerged as a promising complement to graphene. By integrating acetylenic linkages into hexagonal carbon frameworks, graphyne introduces tunable band gaps, directional charge transport, and anisotropic physical characteristics—features that are largely absent in graphene. These structural modifications enable unique electronic, optical, and mechanical responses, positioning graphyne as a potential material for semiconductors, hydrogen storage, membranes for desalination, and energy harvesting devices.

### 1.1. Overview of the Article

This review article comprehensively explores graphyne, a novel carbon allotrope composed of sp and sp^2^ hybridized carbon atoms arranged in a two-dimensional lattice. The article delves into the unique structural forms of graphyne, including α-, β-, γ-, and 6,6,12-graphyne, and their remarkable electronic, mechanical, thermal, and optoelectronic properties. It critically examines theoretical approaches, such as Density Functional Theory (DFT), GW approximation, and Nonequilibrium Green’s Function (NEGF), and experimental methods for synthesizing graphyne, highlighting their advantages and limitations.

**Figure 1:**
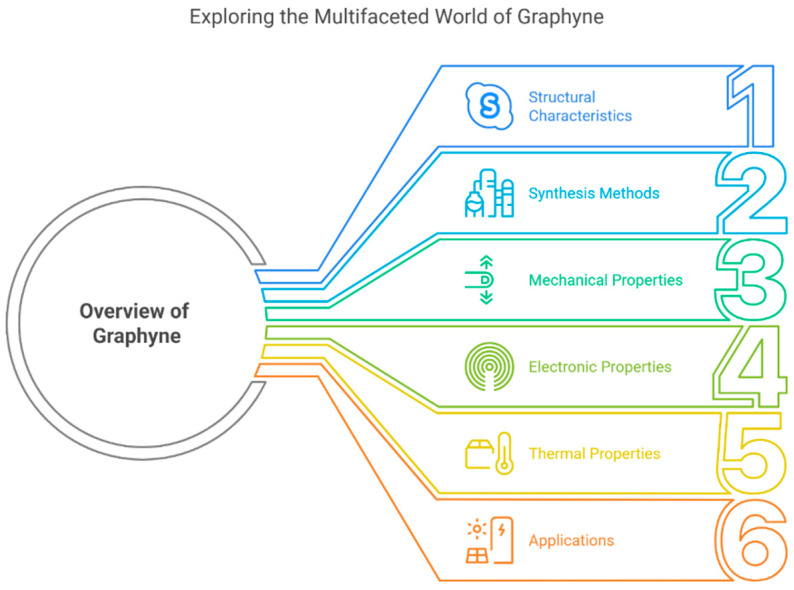
Schematic overview of the review structure, highlighting the relationship between graphyne types, theoretical models, experimental approaches, and application domains.

The review emphasizes how graphyne’s tunable band gap, directional charge transport, and mechanical flexibility make it a promising material for applications in nanoelectronics, energy storage, water desalination, hydrogen storage, and sensors. Despite these promising features, significant challenges remain, including large-scale synthesis, material purity, and integration into practical devices. The article concludes that continued research and collaboration are essential to overcome these challenges and translate graphyne from a theoretical curiosity to a practical technology (Figure 1).

### 1.2. Scope

**Figure 2:**
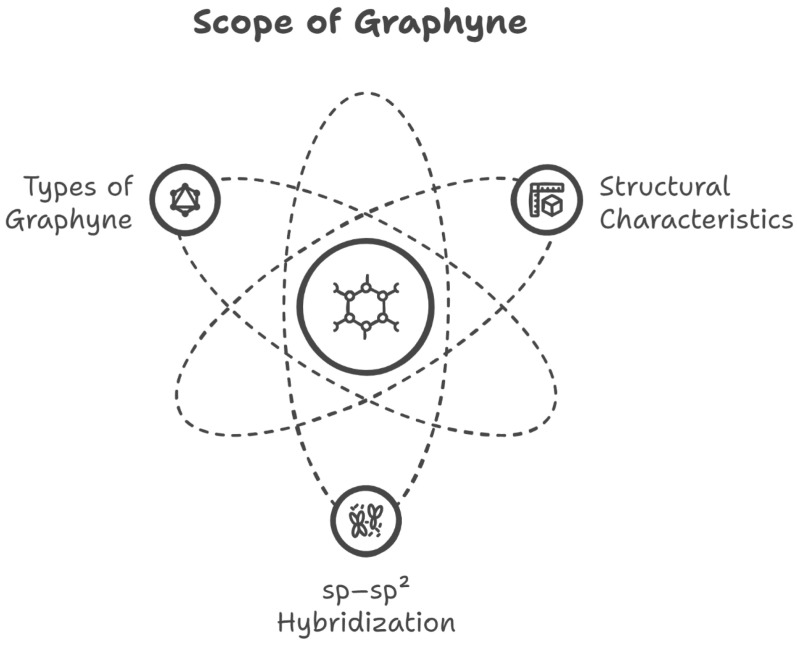
Scope of the review article, emphasizing the integration of structural properties of graphyne with its synthesis techniques and emerging applications.

This article aims to provide a critical and comprehensive review of graphyne’s structural, electronic, mechanical, thermal, and functional characteristics. Unlike conventional reviews, this work integrates theoretical insights from advanced computational models (DFT, GW, NEGF) with experimental developments, offering a balanced perspective on predicted and realized properties. It systematically analyzes various graphyne allotropes (α, β, γ, and 6,6,12) and their influence on fundamental properties, such as band structure, carrier mobility, elasticity, and thermal conductivity. Furthermore, this review evaluates the challenges in synthesis, scalability, and material stability, while identifying emerging application domains, including nanoelectronics, supercapacitors, desalination membranes, hydrogen storage, and sensors. The scope is designed not only to summarize the current state of research but also to outline future directions and highlight critical gaps, positioning graphyne as a versatile candidate in next-generation technologies (Figure 2).

### 1.3. Introduction to Graphyne

GR is a hypothesized carbon allotrope that resembles G but has a distinct arrangement of carbon atoms grouped in a two-dimensional lattice structure. The lattice structure of GR is identical to the honeycomb structure of G, except the C-C bonds are occasionally replaced with -C≡C- bonds. GR can have many lattice configurations because such substitutions can occur at different C-C bonds. The structures of GR are categorized into four types based on the distinct combinations of -C≡C-, namely, α-graphyne, β-graphyne, γ-graphyne, and 6,6,12-graphyne [70].

Except for 6,6,12-graphyne, which has rectangular symmetry, and the initial cell has a rectangular shape with in-plane anisotropy, most GRs have hexagonal symmetry like G. Among them is α-graphyne, which can be created by replacing all carbon-carbon bonds in graphyne with acetylenic (-C≡C-) links. β-graphyne is formed when two-thirds of the carbon-carbon bonds in G are replaced by acetylenic linkages, and γ-graphyne is formed when one-third of the carbon-carbon bonds in GR are replaced by acetylenic links. An α-graphyne has the greatest percentage of acetylenic links (100%). The percentage of acetylenic links in β-graphyne, γ-graphyne, and 6,6,12-graphyne is 66.67%, 33.33%, and 41.67%, respectively [71].

**Figure 3:**
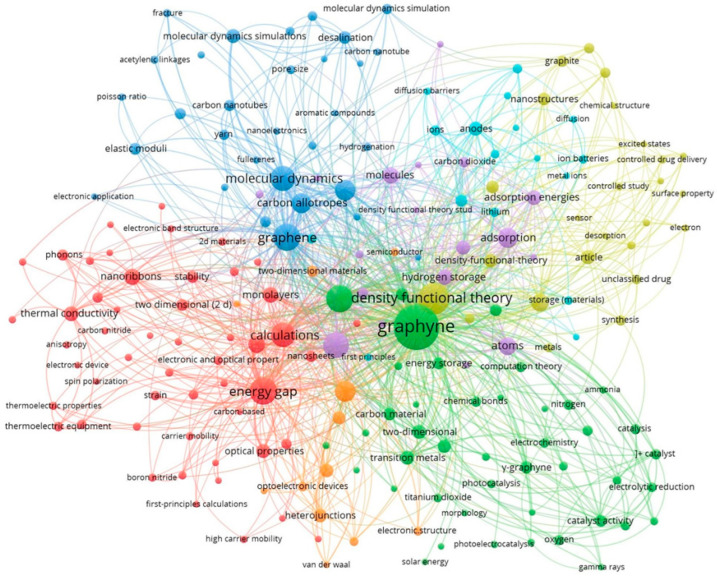
Visualization of the methodological evolution and application areas of graphyne research from 2015 to 2024. (Created by VOSVIEWR).

**Figure 4:**
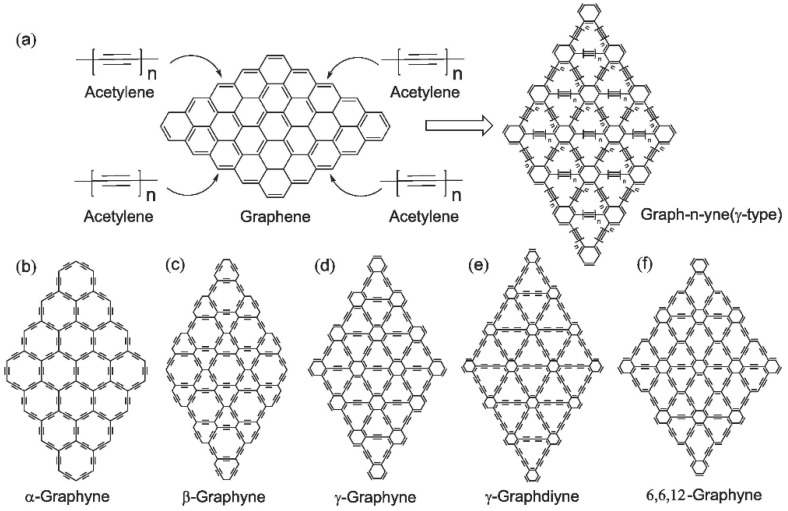
(**a**) Functionalization of graphene to conversion into n-type graphyne. (**b**–**f**) Molecular structures of α-, β-, and γ-graphyne and γ-graphdiyne showing differences in carbon-carbon bonding patterns due to varying acetylenic linkages. Reproduced with permission from reference [77] @ 2024 Elsevier.

Graphyne is an allotrope of carbon consisting of a one-atom-thick sheet of sp and sp^2^ hybridized bonding in the crystal lattice. GR features a two-dimensional crystal lattice and is expected to be synthesized in the future, as only its molecular fragments have been produced so far [72,73]. Due to various arrangements of double and triple bonding, there are discrete types of GR [9,65,74,75]. Because of its structure and applications, a study of graphyne with its application is presented in Figure 3 from 2017 to 2024. Prior to 1960, the existence of GR was only hypothesized. However, following the discovery of fullerene, GR began to attract significant attention from materials scientists [76]. Since 1980, scientists have been working on finding the synthesis method and theoretical properties of the GR. Due to an acetylenic linkage that connects the hexagons, G converts into GR. Compared with graphite and diamond, the GR has a slightly different electronic structure and shows some manifesting properties compared with other materials [65]. There are various types of GR (Figure 4), but γ-GR is known as the common form of GR. The γ-GR has 12 carbon atoms in a unit cell.

In the form of infinite sheets or nano-flakes, GR shows extraordinary mechanical properties that attracted research to investigate them [77,78]. Cranford and Buehler synthesized GR flakes and investigated the mechanical properties by utilizing ReaxFF interatomic potential [77,78]. For accurate values, a time step of 2 fs was considered to calculate mechanical properties against higher frequency [79]. 

Due to the electronic (Dirac cone-like) and mechanical properties of GR, the application of G is limited in field-effect transistors with a high on-off ratio because of its zero-band-gap properties. Additionally, it is used in logic and high-speed switching devices [78,79,80,81]. By utilizing the hydrogenation process, the band gap of the G can be increased [82,83,84,85,86]. By 100% hydrogenation, the G can be converted into GR and is known as a theoretical nonmagnetic semiconductor (SC) with a stoichiometry of CH [87]. Due to the hydrogenation process, the sp^2^ hybridization was converted into sp^3^, and the hydrogen atom changed its direction along the G sheet. In the annealing process, the G can be retrieved from GR [87]. This feature of switching from one state to another makes it a promotable material for various applications like thermal conductivity, magnetization, hydrogen storage, and electronic properties [70,88,89,90]. It can be considered as a future electronic material. The magnificent mechanical properties exhibit that GR can be the future material for mechanical applications compared to other polymer-based membranes.

## 2. Computational Approach

To accurately predict and understand the fundamental properties of graphyne, especially in the absence of widespread experimental data, computational modeling has become indispensable. Among the most employed methods is Density Functional Theory (DFT), a quantum mechanical framework that simplifies the complex many-electron problem by focusing on electron density rather than wave functions. DFT is widely used for calculating electronic band structures, charge distribution, and mechanical responses. However, standard DFT often underestimates the electronic band gap due to its approximate treatment of exchange-correlation energies, which limits its predictive accuracy for semiconducting materials like graphyne.

To overcome this limitation, the GW approximation is employed—named after the Green’s function (G) and the screened Coulomb interaction (W). The GW method provides a more accurate description of quasiparticle energies by accounting for many-body electron-electron interactions that DFT neglects. This is particularly important for materials like graphyne, where precise band gap values and electronic transitions determine their suitability for applications in transistors, photovoltaics, and sensors. For instance, while DFT may predict a small or zero band gap in some graphyne forms, the GW approximation can reveal a nonzero band gap that aligns better with potential semiconductor behavior.

Additionally, tight-binding models are used for their computational efficiency, especially in exploring large or complex graphyne systems. These models use predefined parameters to approximate the electronic structure and are useful for studying trends across different graphyne derivatives. For charge transport analysis, the Nonequilibrium Green’s Function (NEGF) formalism becomes critical. NEGF enables the simulation of current flow in nanoscale devices under bias, making it ideal for assessing graphyne’s performance in electronic applications such as field-effect transistors (FETs).

**Figure 5:**
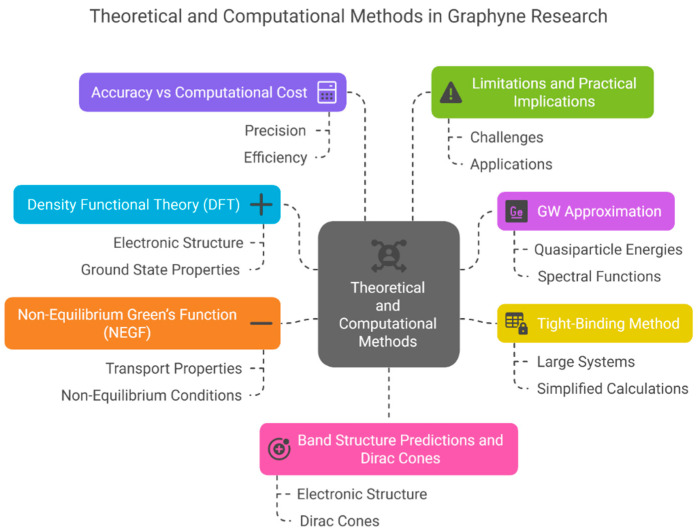
Computational modeling approaches for graphyne analysis, including Density Functional Theory (DFT), GW approximation, tight-binding methods, and Nonequilibrium Green’s Function (NEGF) formalism.

Together, these computational tools provide a multiscale understanding of graphyne’s structure-property relationships—DFT for general electronic and mechanical properties, GW for accurate quasiparticle energy predictions, tight-binding for efficient band structure analysis, and NEGF for transport and device-level simulations. They collectively guide the design (Figure 5), doping strategies, and application potential of graphyne-based materials.

### 2.1. Density Functional Theory

Density Functional Theory (DFT) is a powerful tool that has revealed that the electron density of a many-electron system can determine the properties of this system. In DFT, the many-body problem is transformed into a single-particle Kohn–Sham equation [91,92]. DFT is exact in principle, but the actual form of the exchange-correlation energy term (E_xc_) is unknown. Hence, approximate functionals for E_xc_ are usually used [92,93]. Local density approximation (LDA) assumes that the E_xc_ functional depends only on the value of local electronic density. In contrast, generalized gradient approximation (GGA) considers both the electron density and its gradient. LDA and GGA are widely used to investigate the properties of materials. Still, they underestimate the band gap, resulting from the missing derivative discontinuity of total energy at integer particle numbers. Hybrid functionals, such as HSE [94], mix nonlocal Hartree-Fock exchange with LDA or GGA energy and provide better band gaps than LDA or GGA, but at a higher computational cost.

### 2.2. GW Approximation

The many-body perturbation theory based on the one-body Green’s function is currently considered the most suitable approach to study electronic quasi-particle excitations [95]. In this approach, the nonclassical many-body effects are incorporated by the energy-dependent and nonlocal self-energy term Σxc. The GW method approximates the Σxc by utilizing its first-order expansion concerning the dynamically screened Coulomb interaction W and the Green’s function G [95,96]. The quasi-particle energies are calculated as a first-order correction to the single-particle eigen energies, which are often obtained by computing W and G based on the eigenstates of a reference single-particle Hamiltonian. Although the GW method has been successfully applied to calculate quasi-particle band structure properties for a wide range of materials, it suffers from convergence issues and unfavorable scaling of the computational cost with respect to the system size.

### 2.3. Semi-Empirical Tight-Binding Method

The tight-binding method is a valuable approach for calculating a material’s band structure. It employs atomic orbitals as the basis for expanding the single-electron wave functions of the system. The Hamiltonian matrix elements between these atomic orbitals are considered adjustable parameters [97], which are fitted to experimental or first-principles calculation results. The eigenvalues and eigenvectors are then obtained through the diagonalization of the Hamiltonian matrix. Despite its relative simplicity, the tight-binding model can offer good qualitative results with significantly lower computational costs than DFT calculations. However, its reliance on fitted parameters can result in poor transferability to other systems, thereby limiting its utility in some cases.

### 2.4. Non-Equilibrium Green’s Function Method

The Nonequilibrium Green’s Function (NEGF) formalism is a widely utilized method for determining electron or phonon transport properties of extended systems. This approach involves the construction of a simulated system with two semi-infinite leads serving as electron or heat baths, connected by a central conductor region [98]. The transmission of an electron or phonon is calculated based on the Green’s function for the center region and the self-energy of the leads, which describes the lead-center interaction [99]. The NEGF method offers a distinct advantage. It preserves quantum mechanical effects such as tunneling and diffraction, providing a highly accurate description of nanoscale devices. However, it is worth noting that the NEGF method can be computationally expensive for larger devices.

## 3. Experimental Approach

**Figure 6:**
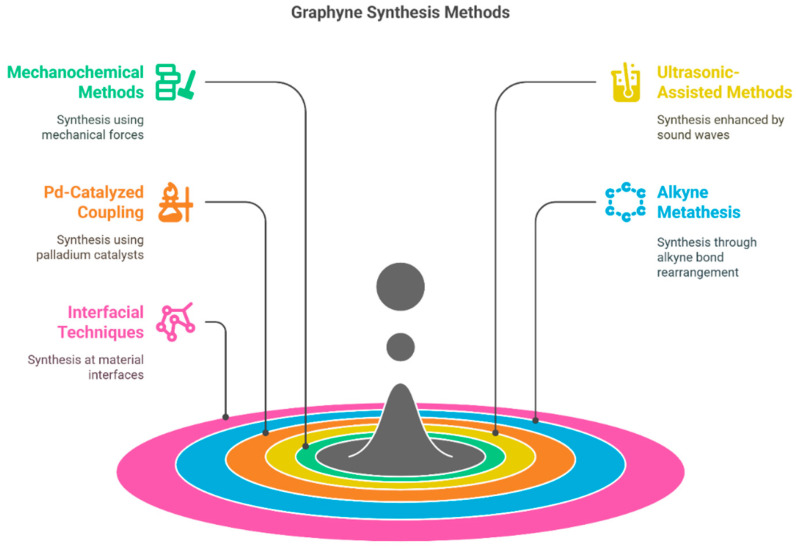
General schematic of graphyne synthesis pathways, including experimental and chemical methods, such as cross-coupling reactions, mechanochemistry, and ultrasonic-assisted synthesis.

The overview of the synthesis process is displayed in Figure 6.

### 3.1. Synthesis of GR

Li et al. produced the first successful process for synthesizing GR in 2010 on the copper surface through the cross-coupling reaction of hexaethynylbenzene [12]. Since then, much progress has been made in the synthesis of GR, and a broad summary of the synthesis techniques for various types of GR is elaborated here.

### 3.2. α-Graphyne

Till today, the synthesis of α-graphyne, a theoretical carbon allotrope, has been a subject of interest, but there were no documented experimental procedures for its synthesis. There could have been additional breakthroughs in the field since then. If scientists have succeeded in synthesizing α-graphyne, the methods would most likely include manipulating and assembling carbon atoms into the particular acetylenic bonds and hexagonal carbon rings that characterize α-graphyne. Chemical vapor deposition, molecular self-assembly, and other advanced synthetic processes could be used to create this unusual carbon allotrope.

### 3.3. β-Graphyne

β-graphyne, like α-graphyne, had not been synthesized experimentally [68,69,100]. β-graphyne is also a theorized carbon allotrope with distinct structural features that include acetylenic bonds and hexagonal carbon rings. Researchers were mostly focused on theoretical studies at the time [101], and experimental synthesis procedures had not yet been devised.

### 3.4. γ-Graphyne

**Figure 7:**
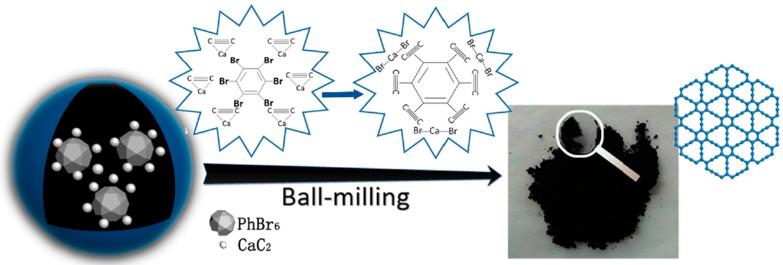
Mechanistic diagram of γ-graphyne synthesis using CaC_2_ and hexabromobenzene via ball milling, illustrating bond formation and substitution steps during the mechanochemical reaction. Reproduced with permission from reference [102] @ 2022 Elsevier.

**Figure 8:**
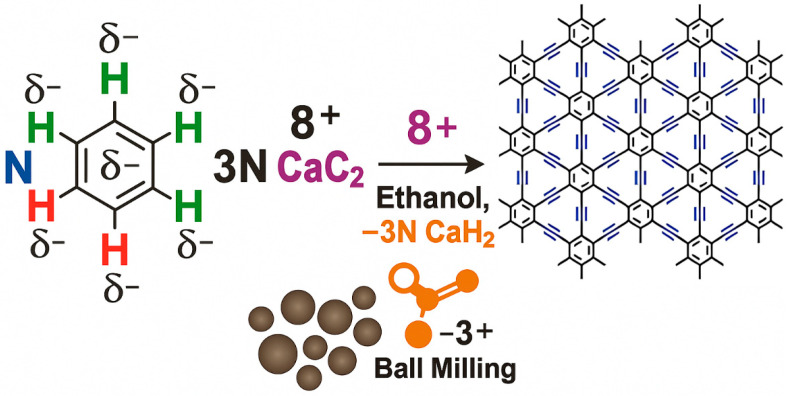
Schematic of γ-graphyne synthesis from benzene and CaC_2_ under a solid–liquid mechanochemical interface reaction, used for electrocatalytic applications.

**Figure 9:**
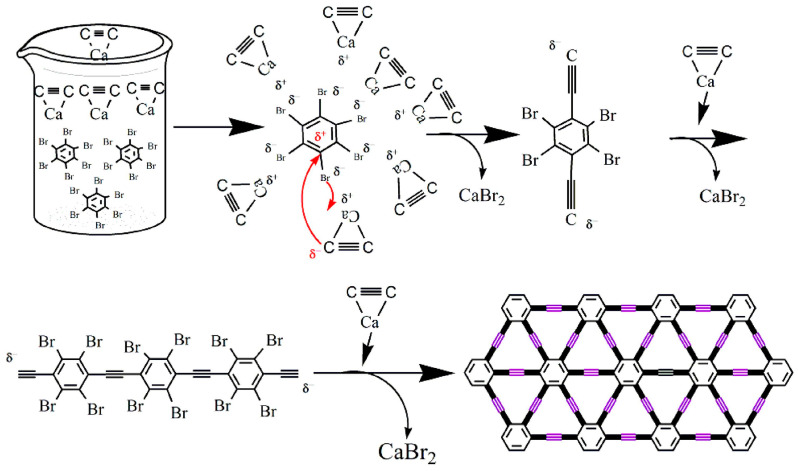
Proposed reaction mechanism for the sonochemical synthesis of γ-graphyne, highlighting the acoustic cavitation-driven activation and enhanced molecular interaction. Reproduced with permission from reference [104] @ 2020 Elsevier.

After the theoretical prediction of γ-graphyne, lots of successful methods, such as mechanochemical, ultrasonic methods, ball milling, catalyzed coupling reaction, etc., have been adopted for the synthesis. Some interesting, applied techniques, along with possible mechanisms, have been demonstrated here. For example, Yang et al. [102] synthesized γ-graphyne using a modified mechanochemical technique and then used it as an adsorbent to clean wastewater dyes. The synthesis process used calcium carbide (CaC_2_) and hexabromobenzene (PhBr_6_) as precursor materials and employed the ball milling method for the synthesis of γ-graphyne (as presented in Figure 7). According to them, initially, high mechanical stress breaks the lattice structure and molecular bonds of CaC_2_, producing an abundance of [C≡C]^2−^ and Ca^2+^ in the medium. Then, highly negatively charged [C≡C]^2−^ having strong surface energy reacts with carbon atoms of PhBr_6_, leading to the formation of C-C bonds between them. Later, Br atoms in the PhBr_6_ were gradually ousted by C≡C bonds. Furthermore, partially substituted hexabromobenzene retains its high activity due to the electron-withdrawing impact of C≡C groups, and cross-coupling nucleophilic substitution persists. Ultimately, acetylene bonds replace every Br atom to create γ-graphyne. In another work, Li et al. [103] also synthesized γ-graphyne through a similar mechanochemical method using benzene as a precursor material. They convert benzene into γ-graphyne by reacting it with CaC_2_ through solid-liquid interphase reaction in the presence of ball milling (Figure 8). The prepared materials were used as electrocatalysts for the oxygen evolution reaction (OER). The authors estimated the Gibbs free energy change in this synthesis reaction and found that it is below zero at a standard temperature, demonstrating a spontaneous process in thermodynamics. Ding et al. [104] also synthesized γ-graphyne using CaC_2_ and PhBr_6_ as a starting material, but they employed ultrasonic force instead of ball milling as a driving force for the cross-coupling reaction. The plausible steps of the reaction are schematically presented in Figure 9 (Red arrows in the figure represent nucleophilic substitution.). The reaction pathway in the ultrasonic method is similar to that of the synthesis of γ-graphyne ball milling, as mentioned previously. Here, only instead of ball milling forces, ultrasound irradiation may rapidly disperse big particles of raw materials into small sizes, considerably increasing the contact area of the reactants. Meanwhile, the high energy given by ultrasonic irradiation’s acoustic cavitation can fully initiate the chemical interaction between CaC_2_ and PhBr_6_. In another work, Yang et al. [105] employed mechanochemistry to synthesize γ-graphyne by cross-coupling reaction utilizing calcium carbide and 1,3,5-tribromobenzene as starting material.

**Figure 10:**
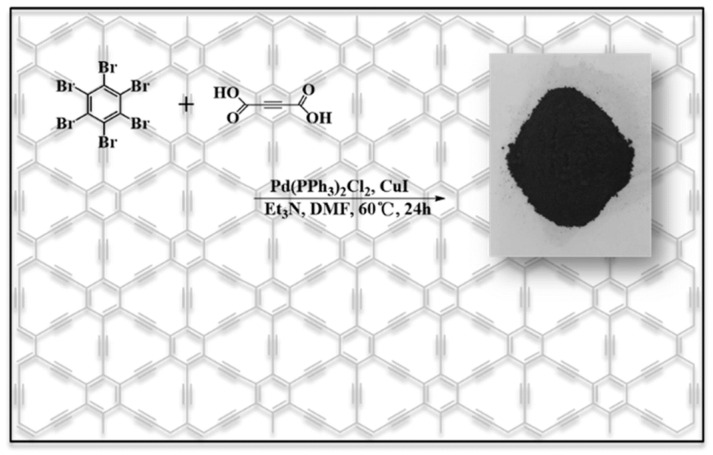
Catalyzed coupling reaction route for γ-graphyne preparation using hexabromobenzene and acetylenedicarboxylic acid in a Pd-catalyzed one-pot reaction. Reproduced from reference [106].

Recently, He et al. [106] employed a one-pot pproach for the synthesis of γ-graphyne utilizing hexabromobenzene and acetylenedicarboxylic acid through a Pd-catalyzed decarboxylative coupling process (as presented in Figure 10). Without a particular substrate or template, the reaction conditions are simple to manage and inexpensive. Compared to previously discussed mechanical ball milling or ultrasonic-assisted synthesis methods, it could obtain γ-graphyne in high yield with minimum equipment requirements, successfully meeting mass production needs. Later, Pd nanoparticle clusters were decorated over the synthesized γ-graphyne sheets, which exhibit excellent catalytic activity towards the reduction of 4-nitrophenol (4-NP) because of the synergistic effect of Pd nanoparticles with the high surface area of γ-graphyne.

**Figure 11:**
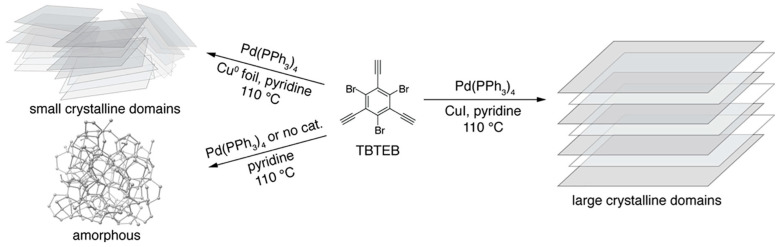
Effect of modified Sonogashira coupling conditions on the crystallinity of γ-graphyne synthesized from 1,3,5-tribromo-2,4,6-triethynylbenzene (TBTEB), demonstrating improved flake morphology with catalyst-assisted polymerization. Reproduced from Reference [107] @ 2022 ACS.

In another work, using 1,3,5-tribromo-2,4,6-triethynylbenzene (TBTEB), Tetrakis(triphenylphosphine) palladium(0) [Pd(PdPh_3_)_4_], and pyridine as the monomer, catalyst, and solvent, respectively, Desyatkin et al. [107] synthesised multilayer γ-graphyne through the Sonogashira coupling reaction. TBTEB broke down in heated pyridine without the need for catalysts, producing an amorphous carbonaceous substance. The TBTEB in hot pyridine was treated to a stoichiometric quantity of Pd(PdPh_3_)_4_ and a Cu foil, resulting in a black, glossy material with submicron-sized crystalline domains oriented randomly. Upon utilizing Pd(PdPh_3_)_4_ and copper iodide (CuI), the reaction produced hexagon-shaped flakes of γ-graphyne, indicating significantly increased crystallinity (as demonstrated in Figure 11). The γ-graphyne produced through the Sonogashira coupling reaction has better quality and purity in comparison to the mechanochemically synthesized γ-graphyne.

Liu et al. [108] synthesized holey graphyne (HGY) from 1,3,5-tribromo-2,4,6-triethynylbenzene as a monomer through a Castro–Stephens-type coupling reaction. For the synthesis of HGY, the interface technique between two immiscible liquids was employed. Copper acetate (CuOAc) and pyridine were present in the upper aqueous solvent layer. They served as a catalyst for the acetylenic homocoupling reaction, and the monomer was present in the lowest organic layer of dichloromethane (DCM). The reaction was then able to avoid the catalyst and monomer chance contact by adding a buffer layer of water in the middle portion, and the carbon-carbon coupling reaction at ambient temperature was conducted for 48 h in an inert argon environment for the synthesis of HGY. Additionally, alkyne metathesis is another method for the synthesis of γ-graphyne. Using this method, the synthesis of γ-graphyne was suggested employing 1,2,3,4,5,6-hexapropynylbenzene (HPB) as a monomer, but the first attempts failed due to the difficulties of the fabrication of an efficient catalyst [109]. Later, Hu et al. [110] created an efficient process via alkyne metathesis to produce highly crystalline bulk γ-graphyne. They were first trying to prepare γ-graphyne from HPB monomer only, and they obtained an amorphous solid with undesired side products. Then, 1,2,3,4,5,6-hexakis [2-(4-hexylphenyl) ethynyl]benzene (HHEB) was used as a comonomer with HPB, and they were very successful in the synthesis of highly crystalline γ-graphyne. According to them, HHEB significantly improved the solubility of γ-graphyne oligomers, allowing them to develop into larger ordered domains prior to precipitation, leading to highly crystalline material [110,111]. Until now, few methods have been developed for synthesizing γ-graphyne, and the developed methods have some limitations, such as complex methods, expense, time consumption, low yield, etc. [111,112]. Thus, there is still a need to create more straightforward and economical processes for producing γ-graphyne in large quantities.

### 3.5. 6,6,12-Graphyne

6,6,12-graphyne has been only theoretically investigated, and still now, experimentally, it has not yet been synthesized. At the time, like α-graphyne and β-graphyne, scientists primarily concentrated on theoretical investigations, and experimental synthesis processes had not yet been developed. Researchers are continuously working on developing synthesis methods for α-graphyne, β-graphyne, and 6,6,12-graphyne, and we can expect that soon, the synthesis method will be explored along with their characteristic properties and applications.

## 4. Properties of GR

**Figure 12:**
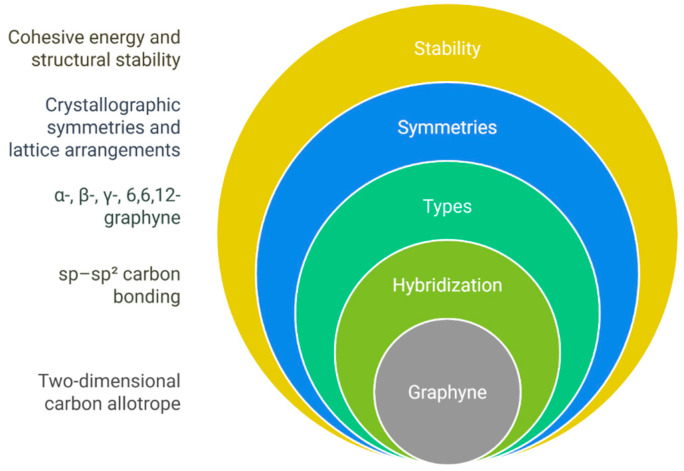
Graphical representation of graphyne’s core physical properties, including structural symmetry, band gap, thermal conductivity, and mechanical flexibility.

The properties of graphyne are presented in a graphical Figure 12.

### 4.1. Structure

**Figure 13:**
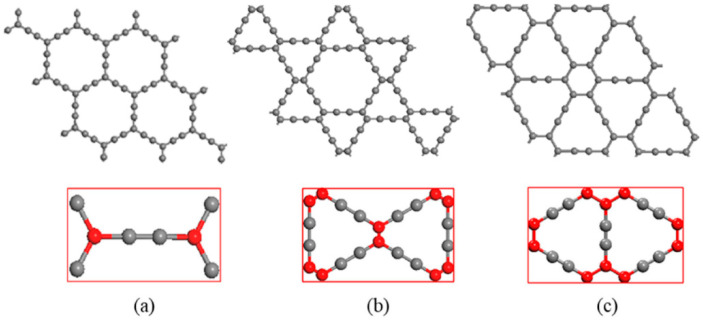
Crystal structures of (**a**) α-graphyne, (**b**) β-graphyne, and (**c**) γ-graphyne showing variations in hybridized carbon bonding (sp/sp^2^) and lattice symmetry. The figure is adopted from Ref. [113] @ 2018 MDPI.

The structure of GR consisted of sp and sp^2^ hybridized rings in the hexagonal lattice structure (Figure 13). Its symmetry looks like G, but the length of the acetylenic linkage can be different. GDR (GR-2) is the first member of this group that was experimentally synthesized [12]. GR-n (in which n indicates the presence of a -C≡C- bond) shows a lower stability because of the insertion of acetylenic linkage (Figure 13a,b) in the carbon hexagonal structure, which causes a reduction in the cohesive energy [9].

**Figure 14:**
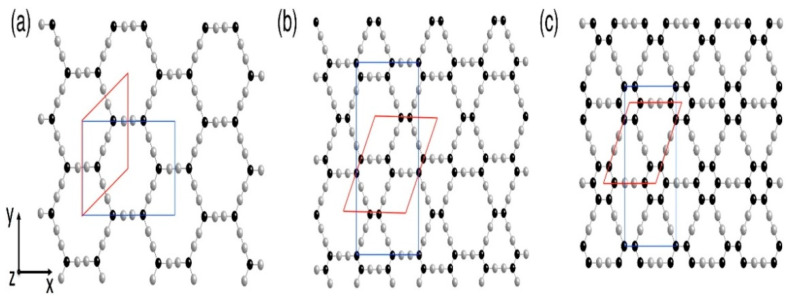
(**a**,**b**) Elastic modulus calculations of graphyne sheets in armchair and zigzag directions using two modeling approaches: (**c**) virial stress (left) and energy minimization (right). Carbon atoms with sp and sp^2^ hybridization are represented in grey and black balls, respectively. The rhombohedral unit cell is highlighted in red, while the rectangular unit cell is shown in blue. Reprinted with permission from reference [114] @ 2016 Elsevier.

The energy reduction in the unstrained scheme shows a constant value, with link lengths of 1.48 Å for single bonds, 1.49 Å for aromatic bonds, and 1.19 Å for double bonds [51]. The study used two different approaches to calculate the moduli of armchairs and zigzag sheets of material Figure 14.

The first approach [115] involved straining the sheet at a constant rate and calculating the virial stress for the interior volume of the sheet to avoid any boundary effects. Using this approach, the armchair modulus was found to be 532.5 GPa, and the zigzag modulus was found to be 700.0 GPa. The second approach [116] involved energy minimization, which gave different results of 629.4 GPa and 772.0 GPa for the armchair and zigzag moduli, respectively. The difference in results obtained from the two approaches was significant.

With the use of the modulation potential “Adaptive Intermolecular Reactive Empirical Bond Order (AIREBO)” (the potential that can be used to make or break the bonds or has the ability to make bonds modulate at various orders), Zhang et al. synthesized GR by molecular dynamics (MD) [117]. Cranford and Buehler used the ReaxFF method to calculate the ultimate stress of GR in zigzag and armchair directions and calculated 49.78 GPa and 46 GPa, respectively [115]. Compared with Cranford and Buehler [115], Zhang et al. calculated the ultimate stress of 63.17 GPa in the armchair direction instead of 46 GPa and in the zigzag direction 49.78 GPa instead of 104 GPa. This difference is observed due to the selection of the calculation method. Cranford and Buehler utilized the ReaxFF method while Zhang et al. used the AIREBO scheme. The ReaxFF method is much more accurate than AIREBO because ReaxFF uses data from the first principle of quantum mechanics, and it has a more accurate description of atoms’ interactions with respect to AIREBO. Still, this computational technique is much more expensive than AIREBO [117]. It has been observed that Zhang et al. calculated their values with a finite-size sheet called “ribbon” in contrast to the infinite sheet considered by Cranford and Buehler [115].

### 4.2. Electronic Properties

**Figure 15:**
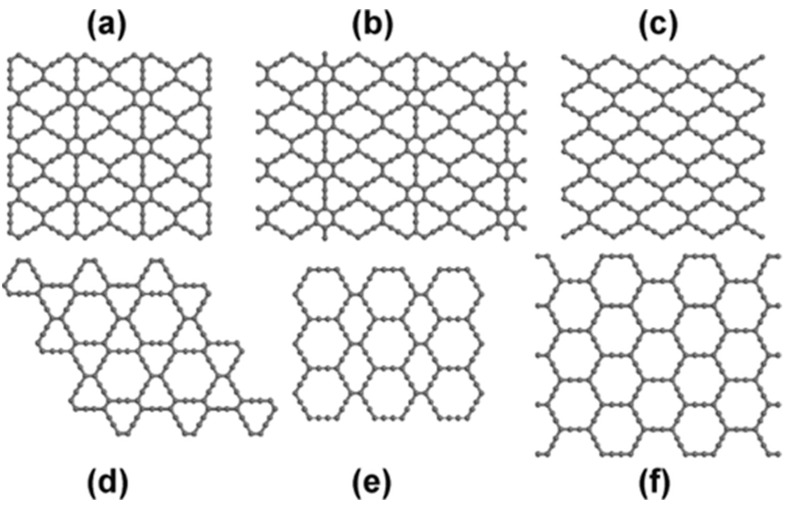
The structures of α-, β-, and γ-graphynes include: (**a**) 6,6,12-graphyne, (**b**) 6,6,14-graphyne, (**c**) 14,14,14-graphyne, (**d**) 12,12,12-graphyne, (**e**) 14,14,18-graphyne (also referred to as β-graphyne), and (**f**) 18,18,18-graphyne (commonly known as α-graphyne). The figure is adopted from Reference [119] @ 2015ACS.

The electronic properties of the GR were calculated through the finite width of nanoribbons with the first-principles method (Figure 15), and it is noticed that the GR falls in the SC group with the band gap range of 0.59 to 1.25 eV [118]. The width of the calculated ribbons was 1 repeat unit to eight repeat units [118]. In the case of infinite sheets, the mechanical strain affects the band gap of GR [56]. It has been observed that the total value of the band gap increased 1 eV by increasing the tensile strain of 0.15 and decreased by 0.3 eV by reducing 1 compressive strain [56].

In GR, the presence of double- and triple-bonded carbon atoms shows a potential for a Dirac cone. Because of this cone, a linear way is observed in the atomic structure. At the Fermi level, conduction and valence bands are overlapped and show a linear fashion. Because of this scheme, in case of no mass, electrons behave as energy, which is directly proportional to the momentum of electrons. GR has the same properties as G, and it shows independent electric properties against the direction of electrons. It is just because of the 6, 6, 12 symmetry in rectangular shape. The electrical grating is observed at the nanoscale due to the dependency of directional symmetry of 6, 6, 12. By controlling this directionality, GR is a considerable material for faster transistors and electronic components in a one-way current process.

The previous study provides information on the changes in chemical properties due to transition metal doping on GR sheets [120]. GR changes its SC properties to spin-polarized metal properties when adsorbed with iron and chromium [120]. Furthermore, a narrow band gap or a spin-polarized half SC behavior was noticed while adsorbed with other transition metals. This property confirms that the transition metals are absorbed on the sheets of GR. Studies have been conducted by researchers to analyze the dielectric real and complex function of the materials that provide information about energy adsorbed in parallel and perpendicular directions [56]. Previously there were two cases studied and a 9-eV mark was used to find the difference among parallel and perpendicular electric fields [56], (i) If the energy is below 9 eV then the parallel electric field shows the greatest response against sensing and (ii) if above 9 eV then the perpendicular electric field shows the greatest response against sensing.

### 4.3. Mechanical Properties

The two main parameters for elastic properties in 2D-type materials are in-plane stiffness and Poisson’s ratio. They are represented by C and ʋ, respectively, and by utilizing the below-mentioned equation. The value of C can be calculated.

(1) $$C=(\frac{1}{S_{0}})\left[\frac{\partial^{2}E}{\partial \varepsilon^{2}}\right]$$

S_0_, ε, and E represent the equilibrium area, uniaxial strain, and total energy of the system [87]. This explains how much a 2D material can tend along an axis and the value of deformation in the direction of the axis. ʋ shows the relationship between transverse strain and uniaxial strain and is represented by

(2) $$\nu =-\frac{\varepsilon_{trans}}{\varepsilon_{axial}}$$

For the hexagonal structure, the isotropic parameters are used, but for the structures 6,6,12-GR, the anisotropic parameters have been adopted. GR has a smaller stiffness with respect to G, see Table 1.

This can be explained with two terms: (1) In G, every C atom has a coordination number N_C_ of 3, and in GR, acetylenic linkage reduces the coordination number N_C_. Due to a small coordination number, a smaller number of atoms are present in the GR. (2) GR has a smaller atomic mass density in comparison with G. The calculation of stiffness of the GR-*n* structure was proposed by Buehler et al., and a linear model scaling was proposed in the study [116]. They proposed a formula [116].

(3) $$C_{n}=C_{1}(\frac{a_{1}}{a_{n}})$$

where C_n_ is utilized to represent GR in-line stiffness and a*_n_* is showing lattice constant. In another study, Fonseca proposed that the mass density is proportional to in-plane stiffness C~$\rho^{p}$ [124]. The Poisson’s ratio for GR is from 0.39 to 0.8, and these values are much higher in comparison with G. The upper bound value of 2D materials is 1.0 [131], and it means that the material cannot change its area under uniaxial strain. The value of 0.87 for α-graphyne may result in the ability to preserve its sheet area.

With this effect, by changing the properties of mechanical strength, the chemical properties changed their behavior, so it is noticed that by changing the mechanical properties, chemical studies have been explored. Because of the -C≡C- presence, the GR shows a lower value of strength with concern to G. On the other hand, GR has a higher ultimate strength value against common polymer-based membranes. In Table 2, various values of the graphene family are presented. In the zigzag direction, GR shows a higher value, while in the direction of the armchair, GR shows a lower value of ultimate strength. The ultimate strength of the GR is dependent on acetylenic linkage and is proportional to the % of -C≡C-, and a scaling law is also proposed for ultimate strength [116]. It is analyzed that the fracture process in the sheet starts from the fracture in C≡C- linkage due to the weak bonding between carbon atoms [117]. The GR also presents extended net strains in the range of 10%, hence, they can hold the artifact under a large strain.

While calculating the elastic energy density, the methodology of the energy minimization method stretched the sheet by a particular amount and ran the methodology on the stretched system. This methodology of calculation of elastic energy density was adopted due to the strain rate because this system is not contingent on the strain rate. In order to calculate the elastic energy density, the energy minimization approach stretched the sheet to a predetermined degree and then minimized energy on the strained system. This methodology is not dynamically true because the dynamically available system cannot calculate the density of the non-stretched GR. The approximate values are 104 GPa and 46 GPa for zigzag and armchair directions, respectively [115]. This difference in values demonstrates Young’s modulus dependence on the strain rate; moreover, the similarity of the net stresses shows that it does not impact the breakdown capability of the material.

### 4.4. Band Properties

**Figure 16:**
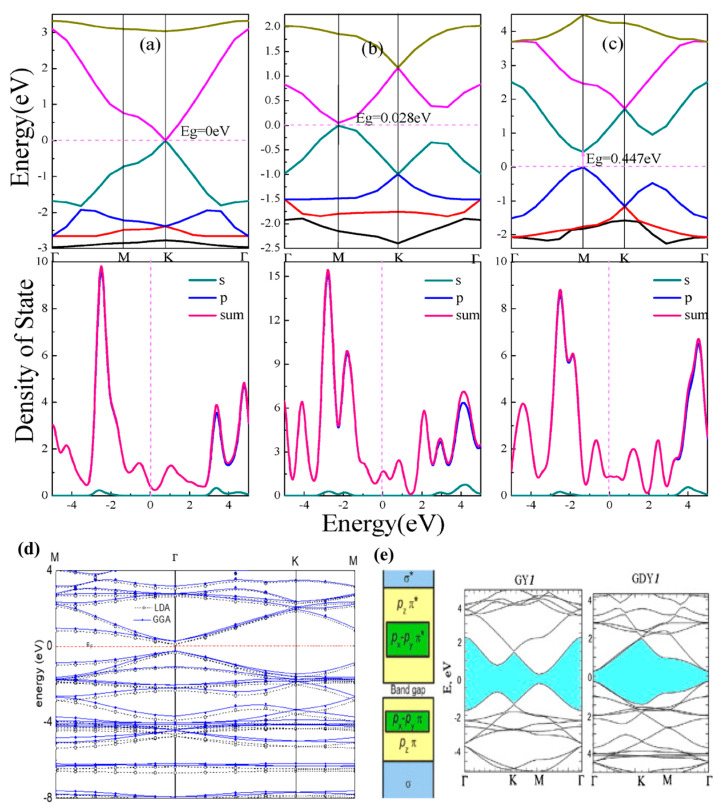
(**a**–**c**) Electronic band structures of α-, β-, and 6,6,12-graphyne. The figure is adapted from reference [113] @ 2018 MDPI. (**d**) Band structure of γ-graphyne calculated using HSE and GGA methods showing direct band gap nature. The figure is adapted from reference [134]. (**e**) Projected electronic states of sp and sp^2^ carbon atoms The figure is reprinted with permission from reference [49] @ 2013 Elsevier.

GR possesses a nonzero band gap that is not present in G [55,56,132]. By utilizing LDA and GGA approaches (Figure 16a), the band gap of graphyne-*n* was calculated to be about 0.5 eV, and it shows a small dependence on acetylenic linkage [55,56,122]. The HSE or GW approaches that have self-interaction correction were utilized to calculate the band gap of GR with the comparison of silicon, and the calculated value was ~1 eV [56,122,132,133]. It has been observed that GR allows a high radiative recombination rate over Si and a light absorption rate over Si because of the direct band gap. From DFT results, the location of the conduction band minimum and valence band maximum was calculated, and it has been located at the M point of the Brillouin Zone of the hexagonal structure. To understand the projected band structure of graphyne, it can be divided into two parts, i.e., sp^2^ and sp [133]. It can be seen (Figure 16) that at the band edges the wide $P_{z}\pi \;to\;\pi^{*}$, deep-lying $\sigma \;to\;\sigma^{*}$ bands are situated, and the inside $P_{z}\pi \;to\;\pi^{*}$ consists of another band $P_{x}-P_{y}\pi \;to\;\pi^{*}$ [56]. In sp–sp interaction, it originates the $P_{x}-P_{y}\pi \;to\;\pi^{*}$ interaction that is not present in G. This leads to anisotropic optical properties in GR [56]. In the range of band gap energy from 0 to 8 eV, this structure adsorbs more energy when in-plane polarized light falls on the material with respect to out-of-plane polarized light [56].

According to the results of first-principles calculations [65], the Dirac cone is also present in α, β, and γ-graphyne Figure 16e. In α GR the Dirac cone is located at higher symmetry points K and K’, while in β- and γ-graphyne, the Dirac cone is situated at lower symmetry points. It has been observed that in 6,6,12-graphyne the Dirac cone is located below and above the Fermi level, which means that it has the property of self-doping [65,135]. This shows that the Dirac cone can be found at any point, and it is not associated with a hexagonal structure, and also it is not limited to G.

**Figure 17:**
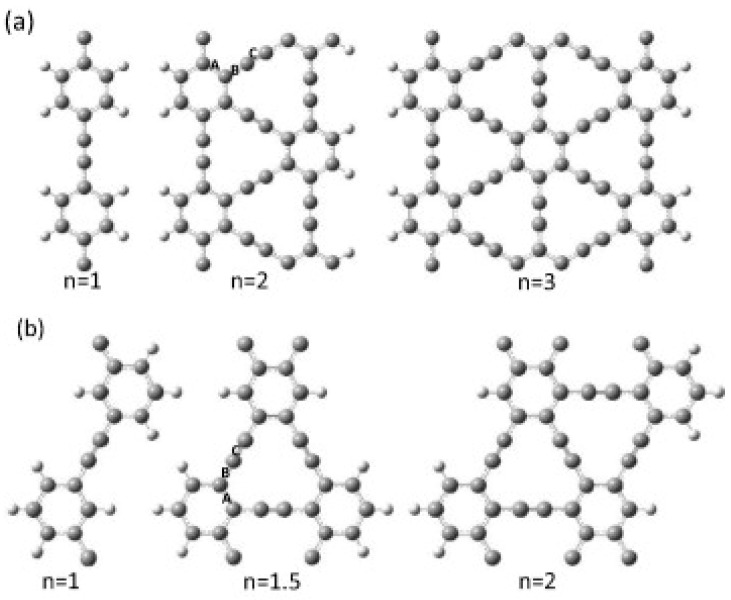
(**a**) Optimized atomic models of armchair-edged graphene nanoribbons (AGNRs) with increasing ribbon widths of n = 1, 2, and 3. (**b**) Corresponding zigzag-edged graphene nanoribbons (ZGNRs) with widths of n = 1, 1.5, and 2, highlighting the variation in edge geometry and periodicity with respect to ribbon width. The figure is adopted with permission from reference [136] @ 2015 Elsevier.

**Figure 18:**
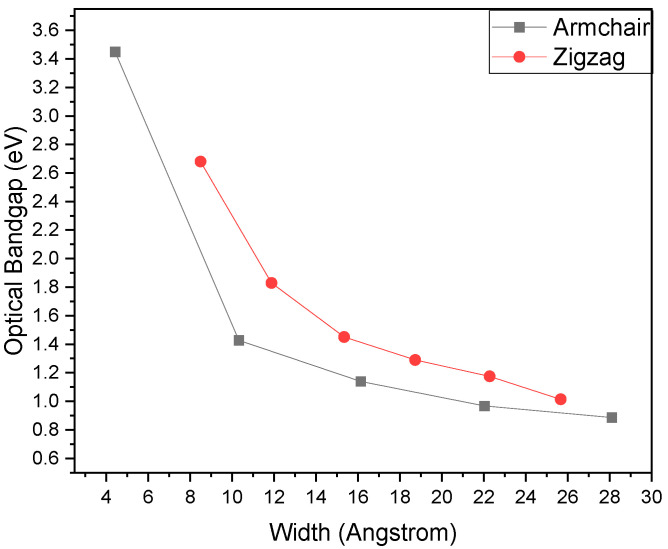
Variation in band gap of graphyne nanoribbons with changing ribbon width, indicating the effect of quantum confinement. Reprinted with permission from reference [136] @ 2015 Elsevier.

Various studies have explored the presence/absence of Dirac cones, and many studies have explored the importance of binding $P_{z}\pi \;to\;\pi^{*}$ [133,135,136,137,138]. This idea proposed that the impact of acetylenic linkage -C≡C- is rival to the renormalized direct hopping term among vertex atoms. Because of this bonding, a tight-binding model can be applied to all types of GR, whereas GR has a hexagonal structure like G. With the existence of Dirac cones, the magnitude and combination of renormalized hopping terms can be determined. By utilizing the above-mentioned model, with the presence of Dirac cones, the new GR-based structures are also predicted. Graphyne 14, 14, 14 has a rhombic symmetry, and it acts the same as deformed G in the direction of the armchair direction (Figure 17). The 14,14,18-graphyne is another that has a rectangular symmetry, and the slope band dispersion shows 5.0 eV/Å on the X-axis and 4.1 eV/Å on the Y-axis with anisotropy linear band dispersion (Figure 18) [133]. A complicated tight-binding model was proposed to check the effect of spin-orbit coupling (C-O-S) on Dirac cones [139]. This model also includes the σ bonding and its effects, which makes it more complicated. By utilizing intrinsic SOC, the nontrivial gap can be opened in α-, β-graphyne. A comparison of graphene, graphyne (α,β,γ) and graphdiyne is presented in Table 2.

**Table 2:** Graphene, graphyne (α, β, γ), and graphdiyne.

Property	Graphene	α-Graphyne	β-Graphyne	γ-Graphyne	Graphdiyne
Carbon Hybridization	sp^2^	sp + sp^2^ [113]	sp + sp^2^ [113]	sp + sp^2^ [113]	sp + sp^2^ [70]
Lattice Structure	Hexagonal	Hexagonal [105]	Hexagonal [105]	Hexagonal [105]	Hexagonal [54]
Acetylenic Linkage %	0%	100% [70]	66.67% [70]	33.33% [70]	Varied (more than γ) [54]
Band Gap	0 eV	~0.44 eV [106]	~0.52 eV [106]	~0.47 eV [106]	0.5–1.1 eV [54,121]
Carrier Mobility (cm^2^/V·s)	~10^5^ [128]	High, anisotropic [128]	High [128]	High (5 × 10^5^) [128]	Moderate–High [129]
Thermal Conductivity	~5000 W/m·K [132]	Lower than graphene [140]	Lower [140]	Lower (~50 W/m·K) [140]	Much lower (~10–20 W/m·K) [140]
Mechanical Strength	~1 TPa [47]	Moderate [78]	Moderate [78]	Moderate [78]	Lower than graphene [106]
Synthesis Status	Scalable [9]	Theoretical [70]	Theoretical [141]	Synthesised [94,95,96,97,98,99]	Synthesised [54,56]
Stability	High	Low (due to 100% triple bonds) [105]	Medium [105]	Medium–High [105]	Medium [54]
Applications	Transistors, sensors, composites [33,34,35]	Theoretical electronics [70]	Theoretical semiconductors [70]	Catalysis, desalination, batteries [94,95,96,97,98,139,142,143]	Sensors, photovoltaics, membranes [54,61,67]

### 4.5. Electronic Transport Properties

G shows the best carrier mobility at 10^5^ cm^2^/Vs [142]. It is suggested that, from theoretical studies, because of extrinsic carrier mobility, GR has some advantage over G from the view of electronic properties. It can be seen that from Table 2, in y-direction 6,6,12-graphyne shows the highest values against all the other structures. By utilizing the Boltzmann transportation equation, with deformation potential theory and relaxation time approximation, Shuai et al. studied the electronic properties of the material [142,143]. This method provides the information that because electron-acoustic phonon coupling at the lower level of energy can cause high carrier mobility, and this mobility is considered as an upper limit of the system. From a device application view, the structure that consists of delocalized electronic states shows an approximated calculation of carrier mobility at 3 × 10^5^ cm^2^/Vs with this method for G [142].

From Table 3, it can be observed that all mentioned structures have considerable carrier mobility in the range of 10^4^ cm^2^/Vs to 10^5^ cm^2^/Vs. Due to the rectangular symmetry, γ-graphyne has obvious anisotropy [142]. The γ-graphyne shows higher carrier mobility in one direction as compared to G, and it shows mobility values of electrons and holes in the range of 5.41 × 10^5^ cm^2^/Vs and 4.29 × 10^5^ cm^2^/Vs, respectively. Because of two Dirac cones in the Brillouin zone with weak electron-phonon coupling can be a cause of this higher mobility [142].

### 4.6. Thermal Properties

The thermal studies were analyzed with the help of various equations and theories such as the Green–Kubo formula with the Boltzmann transport equation, nonequilibrium molecular dynamics, NEGF, and equilibrium molecular dynamics [141,144,145,146,147], GR shows a much lower value of thermal conductivity than what? Previously studied work [141,144,145,146,147] shows that at room temperature, GR has a thermal conductivity of 18–82 W/m K while G shows ~1 × 10^3^ W/m K [146,147]. It has been noticed that as the value of *n* in GR-n increases, thermal conductivity decreases [145,147]. For graphyne-10 the value of thermal conductivity reaches 8 W/m K [145,147] at room temperature. The behavior of the lower thermal conductivity of GR was studied with phonon normal mode analysis [145,147].

**Figure 19:**
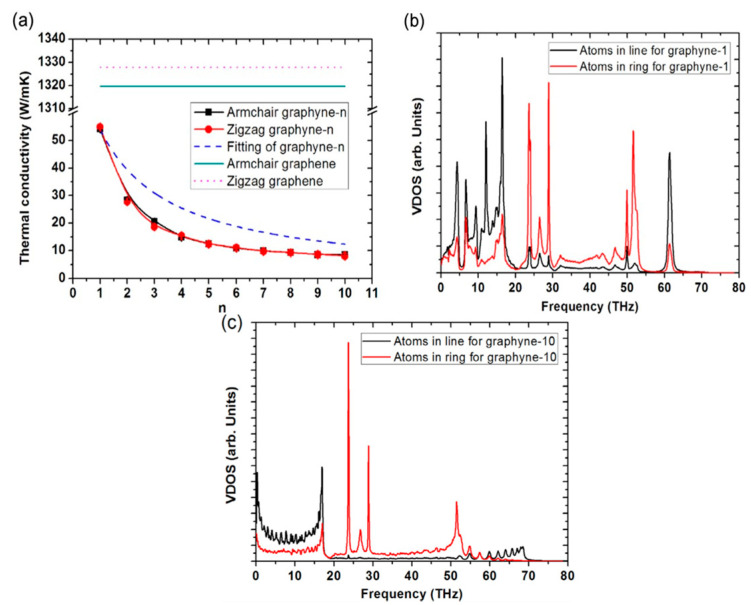
(**a**) Thermal conductivity of graphyne-n and graphene in both zigzag and armchair directions. (**b**) Phonon density of states for GR-1. (**c**) Phonon density of states for GR-10. The figures are adapted with permission from reference [147] @ 2015 Elsevier.

From Figure 19a, it can be seen that due to the combination of linkage atoms to lower frequency phonons, this combination dominates the heat transfer against the atoms present in the hexagonal ring, and as a result, lower thermal conductivity values of GR are observed [144]. The other reason is that due to these combinations, there is a mismatch observed between the hexagonal ring and linkage vibrations and a low value of energy transferred from one part to another. By increasing the linkage length, an enhancement is observed in mismatch vibrations [144,145,146], and a decrement is observed in phonon vibrational density because this density overlaps with the linkage and ring [147]. A higher linkage length causes lower values of thermal conductivity. It has been observed that some low-frequency coherent phonons (f ~ 0 THz) produce the same effect as linkage and ring atoms produced [147]. At lower temperatures (<30 K), the coherent phonons due to excitation produced a greater effect than that of delocalized low-frequency modes in G [145]. The same effect of dependence on linkage is observed in GR nanotubes (GRNT) [148]. The GRNT shows weak dependence on orientation, but in the case of GR nanoribbons (GRNR), because of the edge effects, the orientation can affect the thermal conductivity of GRNR. GRNR demonstrated that the thermal conductivity of the zigzag NR is much smaller than that of the armchair NR even though both have the same width (Figure 19b,c). The anisotropy factor of thermal conductivity is insensitive to temperature. In narrow GRNRs 30% more thermal conductivity was observed as compared to NRs that have a 5-nm width. This difference between GRNRs and GRNT’s exhibited that the edge effect has an important impact on thermal conductance.

### 4.7. Thermoelectric Effect

Thermoelectric outcomes are another interesting property of the GR family. The graphyne-*n* can increase its Seebeck coefficient with a band gap [149]. This can cause a higher thermoelectric performance, which meets the criteria of optimized ZT. The most important thing is that the value of optimized ZT is dependent on the calculation method. At 300 K, with the NEGF method, the values of optimized ZT for the G sheet are 0.16 [149]. With respect to the edge effect, the GYTs show a 3–13 times greater value as related to G NRs [150]. Later, Tan et al. suggested that the values calculated through the NEGF method were overestimated because, in this method, phonon-phonon interactions were neglected and an overestimated value of optimized ZT was calculated [146]. By applying deformation theory with the Boltzmann transport equation, the optimized value of ZT for p-type doping is smaller than n-type doping. At 760 K, the maximum values are 2.92. In another study, Jiang et al. proposed that due to the neglect of phonon scattering in deformation theory, an overestimated value of relaxation time and thermoelectric performance was calculated [141]. To resolve this problem, they utilized density function perturbation theory with the Wannier interpolation technique to calculate the relaxation time of phonon electron interaction and the contribution of all phonons. With the above calculation methods, a peak of ZT was calculated at 0.77 for n-type doping at 600 K [141]. All these studies demonstrated that GR has much better thermoelectric performance compared to G. This effect is also observed in other types of GR, and by introducing defects in β-graphyne, the optimized ZT value can be increased by decreasing phonon thermal conductivity [151].

## 5. Applications

**Figure 20:**
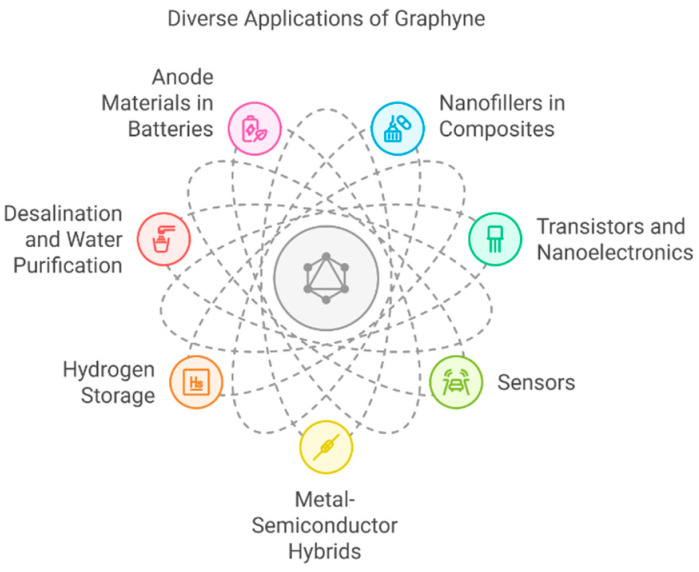
Schematic overview of diverse application areas of graphyne, including transistors, sensors, desalination, energy storage, and catalysis.

The applications of graphyne are presented in Figure 20:

### 5.1. Nanofillers

GR has secured a good score in mechanical properties and is considered the best candidate for nanofillers in composite materials [8,115]. The stiffness and strength can be increased by spreading the flakes of GR on a polymer matrix [8,115]. The literature provides information about the support of GR sheets for the adhesion of fillers on the matrix and increases the effectiveness of the filler by inhibiting the division of the matrix and flakes [8,115]. This forestalls one of the basic unfortunate modes of composites, i.e., the breakup of the scrubby matrix from the utmost performance fiber. Another auspicious indication is the utmost surface adherence energy of GR, 223.5 mJ/m^2^ [8,115], which provides a second mechanism to bind the GR flakes to the matrix. Though GR flakes show anisotropic behavior, mixing into the matrix gives an outcome in the haphazard orientation of the flakes, sharing the resulting composite isotropic properties [8,115]. In this way, GR can be substituted for existing fillers specified as short carbon-based fibers [8,115].

### 5.2. Transistors

For electronic device fabrication, carrier mobility is considered the main part of materials applications. By providing an externally applied field, the high mobility material’s carriers respond quickly and this type of fast response is considered best for high-field effect devices [152]. Computational studies show that the SC behavior of GR can be changed by doping, and the band gap is controllable. It can be used in various transistor applications depending on the band gap, for example, on-off ratio. It is also suggested that by adding GR in conventional SC at the time of fabrication, the various properties of the conventional SCs can be modified, such as on-off ratio, high low voltage control, etc. The numerous properties of GR SCs are dependent on the fabrication size.

### 5.3. Sensors

For various SC devices, electromechanical coupling is a desirable property that can enhance the sensing properties of SCs [153]. For a specific application, mechanical strain can easily modify the material and be easily applied to the material. Because of its high electric strain property, the material can be reshaped after applying the force without permanent deformation. The material can strain again, enabling electromechanical coupling to achieve desirable properties that are good for temperature sensing.

### 5.4. Metal Hybrids SC

GR is proving to be an incredibly useful material in creating narrow-band SCs and spin-polarized half SCs. This is because the SC phase is substantially different from the metal phase, opening up the possibility of making SC metal hybrids on a single monolayer, using GR for both transistors and interconnecting metals in very-large-scale integration fabrication [154]. What’s more, GR’s excellent mechanical properties are a significant advantage in preventing mechanical failure that can result from repeated thermal expansion [115] and contraction that occurs with current technology. Because GR is a single sheet, chemical treatment can be performed as a final step in the fabrication process without the limited penetration that would occur with three-dimensional materials.

### 5.5. H_2_ Storage

**Figure 21:**
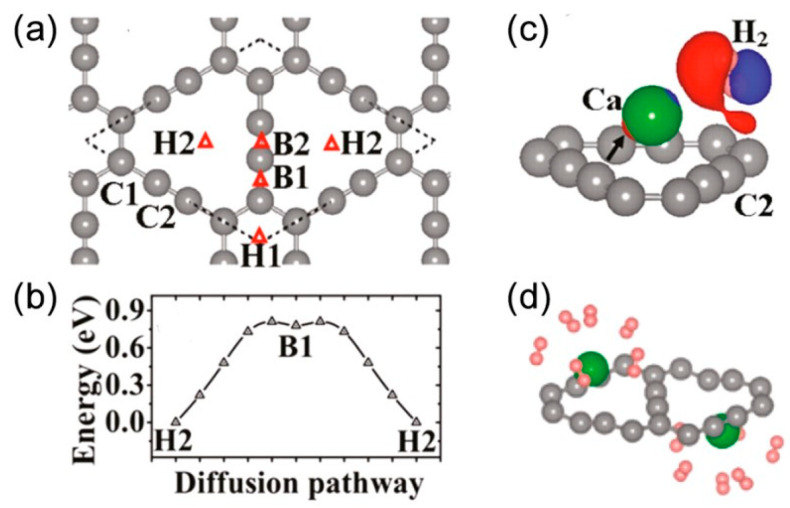
(**a**,**b**) Diffusion path and energy barriers of Ca atoms on γ-graphyne surface for hydrogen storage. (**c**) Charge density difference upon H_2_ adsorption. (**d**) Molecular configuration of H_2_ adsorbed onto Ca sites. The figure is adopted with permission from reference [157] @ 2011 ACS.

Metal-decorated GR, like Ca-decorated GR, has shown promising potential for hydrogen storage. Studies have found that Ca binds strongly to GR due to the presence of the px-pyπ/π* states, and each Ca can bind up to six H_2_ molecules (Figure 21a,b), resulting in a maximum hydrogen storage capacity of 9.6 wt% with an average hydrogen binding energy of ~0.2 eV, which is suitable for practical applications. Moreover, Ca-decorated GRs have been found to be thermodynamically stable. In comparison with G, Ca-decorated G has a lower hydrogen storage capacity of 8.4 wt%, while on the other hand, Li-decorated GR shows higher values w.r.t G [155]. Li-decorated GR is also a promising system for H_2_ storage, as Li has the smallest atomic mass among different metals, and it has a high binding energy with H_2_ (Figure 21c,d). In fact, the maximum storage capacity reported for Li-decorated GR is 18.6 wt%, as shown by Guo et al. [156].

### 5.6. Desalination

**Figure 22:**
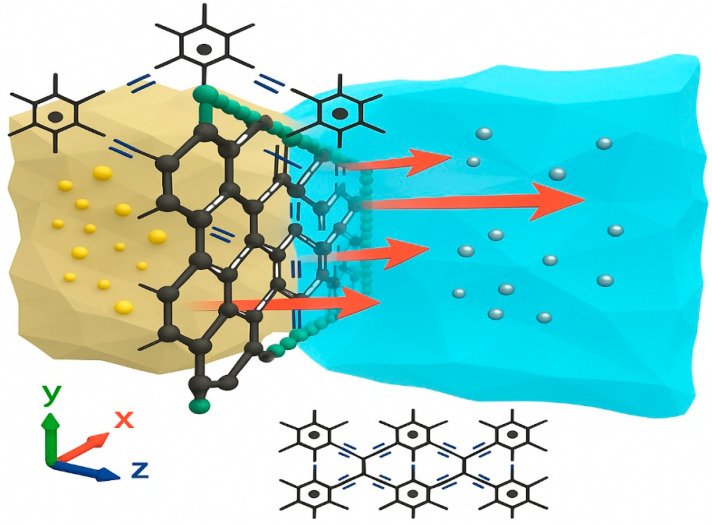
The salination process of GR viewed systematically from a computational perspective, involves allowing water molecules to pass under external pressure.

A total of 97.5% of the global water is saltwater or seawater on Earth [158]. The desalination process can obtain clean water from salt/seawater. This can solve the problem of normal water necessary for industry, drinking, agriculture, etc. The size of holes that are created in GR due to the double and triple bonding of carbon atoms is of the order of chains, and the water molecules can pass through the membrane made of GR. However, it is too small for sodium and chlorine ions surrounded by electrically attracted water molecules (Figure 22).

**Figure 23:**
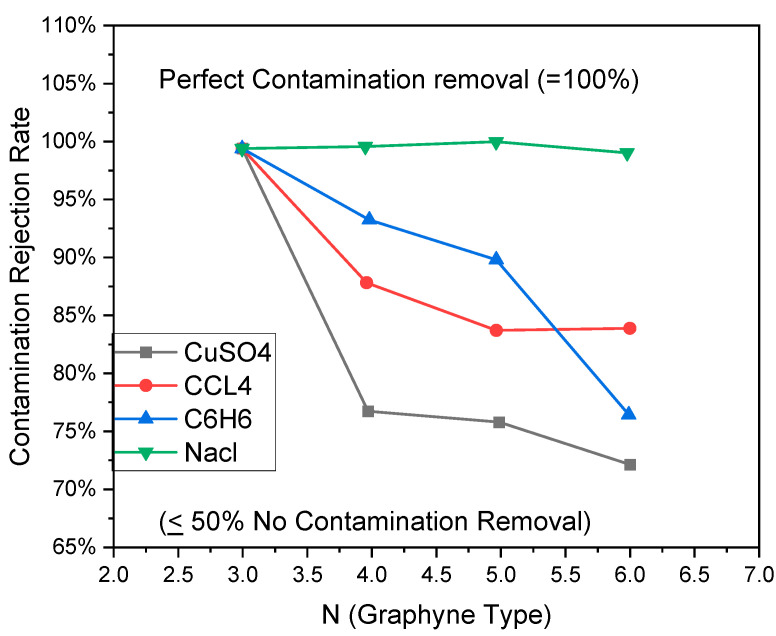
Rejection of various salt ions (e.g., Na^+^, Cl^−^, Ca^2+^, Mg^2+^) by graphyne membranes under a pressure gradient of 50 MPa. Similar kinds of results were published in references [160].

The GR can block approximately 100% of all types of ions (Figure 22), such as Ca^2+^, Na^+^, Cl^−^, K^+^, and Mg^2+^ [159]. The calculations run with the help of the first principle modeling and MD simulations gave roughly two orders of magnitude higher than that of commercialized state-of-the-art turnabout osmosis membranes with a salt blockage of 98.5% [159]. The GR shows the blockage of ions (Figure 23), which is independent of ion concentration and operating pressure. It is suggested that these stopping barriers of GR produce energy barriers that are higher for salt ions than water [159].

### 5.7. Anode Applications

**Figure 24:**
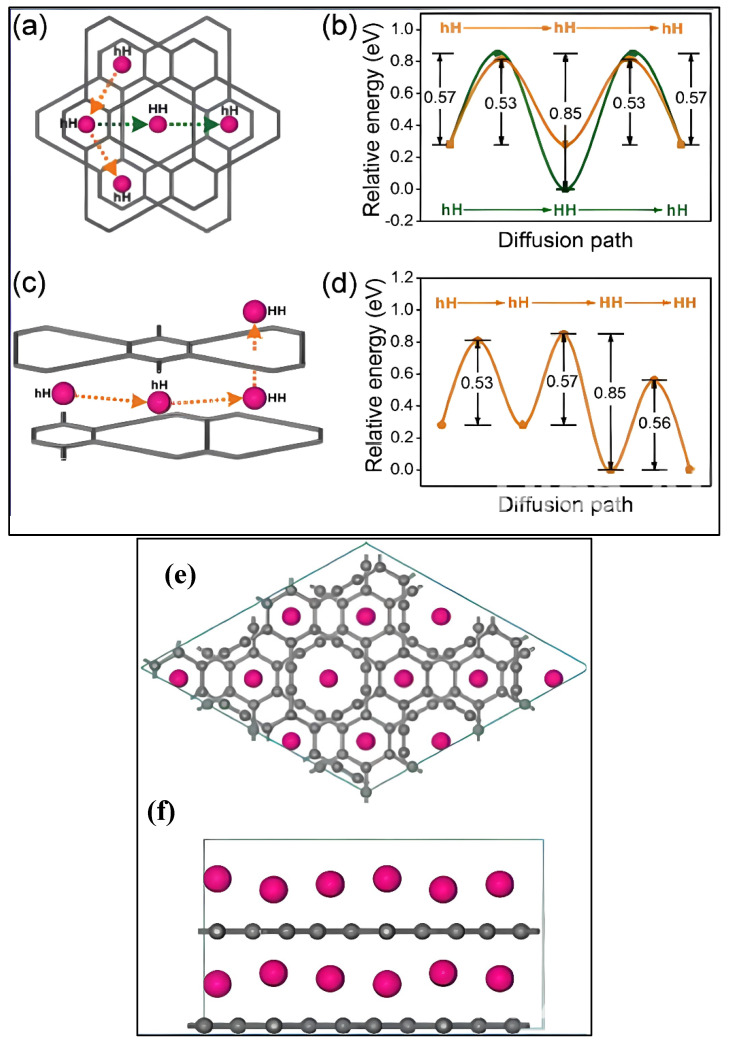
(**a**,**b**) In-plane diffusion pathways of Li ions in bulk graphyne. (**c**,**d**) Out-of-plane diffusion barriers indicating 3D ion mobility for energy storage. Reprinted with permission from Reference[161] @ 2011ACS. (**e**) Top view and (**f**) side view of the optimized atomic configuration of LiC_4_, illustrating lithium atoms intercalated within the graphyne lattice. The arrangement highlights the spatial positioning of Li ions in relation to the sp/sp^2^ carbon framework, with lithium residing both above and within the hexagonal carbon layers. Reprinted with permission from Reference [161] @ 2011ACS.

An ideal anode material for a lithium- or sodium-ion battery requires high mobility and a high storage capacity of ions. Theoretical calculations indicate that GFMs can fulfill these requirements. It has been shown that due to the special atomic structure of bulk GR, three-dimensional Li diffusion can be achieved (Figure 24), and for sodium storage in GR and GDR, the calculated capacities are NaC_4_ and NaC_3_, respectively. The small barrier of about 0.4 eV enables two-dimensional fast Na diffusion on GR and GDR. All these studies suggest that GFMs can have excellent performance as the anode of lithium or sodium ion batteries.

### 5.8. Electrocatalyst

**Figure 25:**
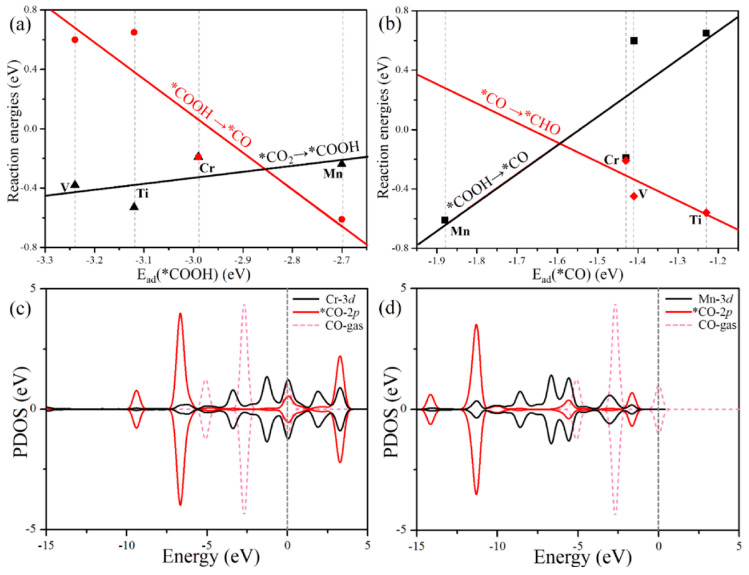
(**a**,**b**) Reaction energy diagrams for *CO_2_ → *CO and *COOH → *CHO conversions on various M-GY catalysts. (**c**,**d**) Projected density of states (PDOS) of CO adsorbed on Cr-GY and Mn-GY, showing interaction between CO states and the metal sites. The Fermi level is set to 0 eV. The figure is adopted with permission from reference [166] @ 2021 Elsevier.

Graphyne has recently garnered attention in electrocatalysis owing to its conjugated π-electron system, high surface area, and tunable electronic properties through doping or structural modifications. Its unique sp–sp^2^ hybridized network introduces active sites suitable for redox reactions, especially when decorated with transition metals or heteroatoms. Among various graphyne forms, γ-graphyne has shown promising activity in oxygen evolution reactions (OER), hydrogen evolution reactions (HER), and carbon dioxide reduction due to its modifiable band structure and favorable adsorption energy for catalytic intermediates (Figure 25).

Advanced computational investigations have demonstrated that graphyne-based materials, when functionalized with elements such as Fe, Co, or Ni, exhibit comparable or superior activity to traditional catalysts [162]. For instance, density functional theory (DFT) calculations reveal that Fe-doped γ-graphyne significantly reduces the Gibbs free energy of hydrogen adsorption, thus enhancing HER kinetics. Furthermore, palladium nanoparticles anchored on γ-graphyne frameworks have shown improved catalytic behavior in 4-nitrophenol reduction, emphasizing the synergistic effect between metal centers and the extended conjugation of the graphyne sheet [163,164].

These findings underscore the potential of graphyne derivatives as next-generation electrocatalysts [165]. Their conductivity, coupled with abundant binding sites and chemical tunability, positions them as strong candidates for sustainable catalysis in energy conversion systems such as water-splitting cells and fuel cells. However, to transition from theoretical insights to practical implementation, challenges related to structural stability, synthesis scalability, and long-term electrochemical performance must still be addressed.

## 6. Summary

The structure of GR can be seen as a two-dimensional (2D) network of hexagonal carbon rings (sp^2^ hybridized) linked by acetylenic connections (sp hybridized). Baughman et al. [9] proposed graphyne as a structure in which one-third of the C-C bonds in G are substituted with one acetylene unit, and it has been proven to be structurally stable and synthetically accessible. Acetylene groups are shown to lower binding energy and modify optical and electrical characteristics in a diverse manner [55,125]. GR has a similar hexagonal symmetric structure to G. The number of acetylenic bonds can vary, resulting in *n* number of GR structures, where *n* is the number of -C≡C- bonds in the linkage. The stability of GR structures is lower than that of G owing to the introduction of acetylenic bonds in the structure, resulting in a lowering of cohesive energy. As the number of acetylenic bonds increases in the G structures, the cohesive energy decreases, leading to a decrease in the stability of the compound [9,167]. G and GR have similar out-of-plane deformation and interfacial adhesion, despite GR having just half of G’s density [112]. In theory, GRs have superior and exceptional electronic characteristics than G because of their natural band gap of 0.44–2.23 eV. The existence of a triple carbon-carbon bond in GR enables reversed chirality features and momentum shifts of their Dirac cones, allowing for tunability of their energy gap [168]. In addition, GR is expected to exhibit remarkable spatial flexibility, low thermal conductivities, and electron transfer capabilities equivalent to G due to its covalent bonding. Additionally, its band structure sets it apart from other carbon allotropes that are currently in use, which makes it a potential material for lithium storage, SC components, and electron mobility, and among other uses, also [169]. Zhang et al. [144] examined the thermal conductivity of four different GRs. They found that it is much lower than G. The acetylenic linkages presence in the GRs led to an excess reduction in thermal conductivity because of the weak single bonds and corresponding low atom density in the structures. The most pronounced directional anisotropic thermal conductivity has been observed in 6,6,12-graphyne, and along with tension and rising temperatures, has a negative effect on the thermal conductivity of GRs. While there are some similarities between G and GR, GRs have a few extra benefits. The chemical structure of 6,6,12-graphyne is especially notable since it demonstrates that the creation of Dirac fermions that resemble G does not depend on the presence of hexagonal symmetry. The directional anisotropy in carrier mobility of the 6,6,12-graphyne indicates its uniqueness [170]. It has been noticed that G adopts a maximum in-plane stiffness. Then, when we move from GR to GDR, this parameter diminishes. As a result, the Poisson’s ratios rise in the same order. The most straightforward explanation for this behavior is that when compared to the most compact structure, G, long acetylenic (and later diacetylene) linkages for G (and subsequently for GDR) cause a progressive structural weakness (a decrease in the atomic bond energy for more loose structures), with the degree of reduction being proportional to the percentage of linkages [117]. Furthermore, the numerical estimates for GR show that independent elastic constants and the Young’s modulus decline by 24.8% and 24.6%, respectively, as the temperature rises from 0 K to 1200 K. These losses are significantly greater than those for G, which only experienced a 3.5% and 4.5% decrease in these parameters. Consequently, the mechanical properties of GR soften noticeably as temperature rises, in contrast to G, which maintains its outstanding mechanical qualities even at high temperatures [49]. With their high mechanical strength, electrical conductivity, and adaptability, GRs, in comparison with their family, hold great potential for groundbreaking developments in electronics, materials science, and energy applications. However, obstacles to the broad commercial use of GRs include issues with scalable production techniques and integration with current technologies.

## 7. Future Prospects

The study of graphyne is still in its nascent stages, and substantial efforts are needed to realize its full potential in practical applications. A primary challenge lies in the controlled and scalable synthesis of graphyne with high structural purity and reproducibility. Although γ-graphyne has been synthesized via various methods, including mechanochemical, ultrasonic, and coupling reactions, these techniques are often hampered by low yields, complex procedures, or prohibitive costs. Future research should prioritize the development of streamlined, low-temperature, and catalyst-efficient methods to produce large-area graphyne films with minimal defects.

From a computational perspective, integrating high-accuracy simulations, such as density functional perturbation theory, time-dependent DFT, and machine-learning-enhanced models, can enhance predictions of band structures, phonon behavior, and defect tolerance across different graphyne allotropes. Moreover, collaborative studies that combine theoretical predictions with experimental validation are crucial for understanding transport properties, such as carrier mobility and thermoelectric efficiency, particularly in doped or heterostructure systems.

Integrating graphyne into devices presents another significant challenge. Embedding graphyne into flexible substrates, hybrid electrodes, or membranes necessitates addressing issues related to interfacial stability, mechanical anchoring, and long-term environmental durability. Emerging application areas, including electrocatalysis, wearable electronics, energy harvesters, and desalination technologies, hold great promise for innovation, provided that material synthesis can meet the performance demands of these fields.

Lastly, the development of functional graphyne-based heterostructures, composites with transition metals, and defect-engineered configurations can unlock properties that are unattainable with graphene alone. Long-term success in this domain will hinge on interdisciplinary collaboration among chemists, physicists, and engineers to surmount existing obstacles and translate graphyne’s unique attributes into viable technologies.

## 8. Conclusions

Below is an insightful review of the latest theoretical advancements in GR’s fundamental properties and potential applications. GR is a remarkably strong material with impressive strain capabilities, making it an incredibly sturdy substance. Depending on how sp and sp^2^ carbon atoms are arranged, GR can be displayed as semiconductors or semimetals with Dirac cones, presenting highly adjustable band structures that can be manipulated through structural engineering, strain, doping, and electric field techniques. GR’s high intrinsic carrier mobility and rich quantum and spin transport properties make it an excellent option for numerous nanoelectronic devices. Although GR experiences lower thermal conductivity than graphene due to vibrational mismatches between atoms in hexagonal rings and acetylenic linkages, it delivers superior thermoelectric performance with a high figure of merit.

Until now, few methods have been developed for synthesizing γ-graphyne, and the developed methods have some limitations, such as complex methods, expense, time consumption, low yield, etc. [8,9]. Thus, there is still a need to create more straightforward and economical processes for producing γ-graphyne in large quantities. Theoretical calculations suggest various applications for GR, including transistors, metal hybrid supercapacitors, water desalination and purification, battery anode materials, and hydrogen storage. Although there are a few challenges, such as the need for experimental validation of theoretical predictions, the development of scalable synthesis techniques, and the exploration of GR’s properties in heterostructures and composites, there is no denying that GR boasts vast potential. It is crucial to maintain continued collaboration between experimentalists and theorists to expedite GR research and realize its potential applications in electronics, catalysis, and energy.

## Figures and Tables

**Table 1:** Various parameters of graphene, graphadiyne, and graphyne family.

**Name**	**Ultimate Strength σ_x_ (N/m)**	**Ultimate Strength σ_y_ (N/m)**	**Poisson’s Ratio v_x_**	**Poisson’s Ratio v_y_**	**In-Plane Stiffness C_x_ (N/m)**	**In-Plane Stiffness C_y_ (N/m)**	**Ultimate Stain ε_x_ (%)**	**Ultimate Stain ε_y_ (%)**
Graphyne	17.84 [121]	18.83 [121]	0.417 [56], 0.416 [122]	0.42 [123]	166 [56], 170.4 [115]	224.0 [115], 169.2	20 [121]	20 [121]
	16.68 [117]	21.16 [117]	0.429 [121], 0.42 [123]	0.38 [124]	166.3 [122]. 162.1 [121]	162.5 [123], 159.6 [124]	11.2 [117]	14.8 [117]
	14.34 [115]	31.97 [115]	0.39 [124]		150 [125], 170.2 [117]		8.19 [115]	13.24 [115]
	14.44 [125]	20.47 [125]			164 [123], 163.0 [124]		11.2 [125]	17.7 [125]
Graphadiyne	10.71 [116]	13.54 [116]	0.446 [122], 0.4 [124]	0.40 [124]	123.1 [122], 150.2 [116]	185.2 [116], 117.5 [124]	6.3 [116]	8.0 [116]
	9.54 [125]	20.84 [125]	0.453		100 [125], 118.6 [124]		10.9 [125]	20.8 [125]
					121.8 [126]			
α-Graphyne	10.88 [117]	12.18 [117]	0.863 [113], 0.87 [114]	0.72 [124]	39.9 [117], 24 [113]	40.2 [117], 42.4 [124]	15.6 [117]	17.8 [117]
			0.874 [127], 0.72 [124]		21.98 [114], 22.48 [127]			
					42.8 [124]			
β-Graphyne	12.75 [117]	15.50 [117]	0.49 [128], 0.647 [113]	0.51 [124]	87.1 [117], 83 [128]	87.4 [117], 92.1 [124]	13.0 [117]	16.2 [117]
			0.67 [114], 0.52 [124]		77 [113], 73.07 [114]			
					93.6 [124]			
6,6,12-Graphyne	13.09 [117]	20.64 [117]	0.39 [124]	0.49 [124]	117.3 [117], 121.1 [124]	149.1 [117], 152.1 [124]	11.6 [117]	14.7 [117]
Graphene	34.71 [117]	41.94 [117]	0.164 [122], 0.169 [129]		347.0 [122], 348 [129], 333 [117]		0.134 [117]	0.191 [117]
	30.15 [130]	35.85 [130]	0.18 [114]		341.09 [114]		0.13 [130]	0.2 [130]

**Table 3:** Mobility of electrons and holes for graphene, graphdiyne, and graphyne family [142].

Name	Direction	μ_h_ (×10^4^ cm^2^/Vs)	μ_e_ (×10^4^ cm^2^/Vs)
Graphene	X	35.12	32.02
	Y	32.17	33.89
Graphdiyne	X	1.91	17.22
	Y	1.97	20.81
6,6,12-Graphyne	X	12.29	24.48
	Y	42.92	54.10
β-Graphyne	X	0.856	0.798
	Y	1.076	0.892
α-Graphyne	X	2.960	2.716
	Y	3.316	3.3277

## Data Availability

Data will be made available upon request.
